# Effects of massage therapy on preterm infants and their mothers: a systematic review and meta-analysis of randomized controlled trials

**DOI:** 10.3389/fped.2023.1198730

**Published:** 2023-08-31

**Authors:** Yu Zhang, Chunlan Duan, Luying Cheng, Haihong Li

**Affiliations:** ^1^ School of Nursing, Lanzhou University, Lanzhou, Gansu, China; ^2^ School of Nursing, Gansu University of Chinese Medicine, Lanzhou, Gansu, China; ^3^ Department of Nursing, Gansu Provincial Maternity and Child-Care Hospital, Lanzhou, Gansu, China

**Keywords:** massage therapy, mothers, preterm infants, meta-analysis, systematic review

## Abstract

**Background:**

Massage therapy for preterm newborns has received increasing attention in recent years due to its beneficial clinical outcomes. However, disagreements persist in different investigations.

**Method:**

We performed a systematic literature search in the Cochrane Library, Embase, PubMed, Web Science, and CINAHL to retrieve randomized controlled trials of premature infants receiving massage therapy and its impact on maternal and infant outcomes. Outcomes were mother-infant attachment, oxygen saturation, motor funtion, reflex, temperature, and calorie intake. The tool developed by the Cochrane collaboration assessed risk bias. With a 95% confidence interval (CI), the integration's results were presented as the mean difference or standardized mean difference. The registration number was CRD42022337849.

**Results:**

Of 940 records retrieved, 15 trials were included. Massage therapy increased oxygen saturation (standardized mean difference (SMD) = 2.00, 95% CI [1.17 to 2.83], *P *< 0.0001). Massage therapy can strengthen mother-infant attachment [SMD = 2.83, 95% CI (2.31 to 3.35), *P *< 0.00001]. Other outcomes, including motor activity, relaxation, caloric intake, and temperature, did not differ significantly.

**Conclusion:**

Massage therapy can significantly improve oxygen saturation and strengthen maternal-infant attachment. However, prior to making a recommendation, additional research with a larger sample size and more rigorous design should be conducted due to the heterogeneity of studies in several outcomes.

## 1. Introduction

More than 1 in 10 babies are born prematurely worldwide each year, which means that there are 15 million premature births each year (1). Preterm birth accounts for an increased risk of mortality and morbidity both in the short and long term (2). In developed countries, advanced maternal age, twin pregnancies, assisted reproduction, and other factors have led to an increase in the rate of premature births (3). Preterm infants and children born with low birth weights exhibit unfavorable growth and development in the future, which is a crucial determinant of the subsequent development of adult diseases (4). While many preterm infants are present in developed countries, mortality for a preterm infant born in a low-income or middle-income country (LMIC) is eight times higher than in Europe (5). Thus, it can be shown that in low- and middle-income countries and regions, there may be a lack of adequate preterm care, which puts the lives of many preterm infants at risk (1). Therefore, early care for preterm infants is extremely important (6).

Several studies have shown the variety of benefits of massage therapy for preterm infants, such as weight gain, immune enhancement, cognitive improvement, neurodevelopment, and shorter hospital stays. Whether as a primary or secondary outcome, weight gain is the most commonly assessed outcome in randomized controlled trials of massage for preterm infants (7). The mechanism of action of massage therapy on weight gain in preterm infants is not completely clear. Related studies suggest that massage may increase the amount of muscle and limb activity, leading to an increase in the infant's metabolism, which promotes the synthesis of glycogen, fat, and protein to increase their body weight (8), and it has been suggested that increased vagal activity induced by massage may lead to increased gastric motility in preterm infants and thus is thought to be a potential mechanism for the greater weight gain in infants receiving massage therapy (9, 10). In a related study, the effect of massage therapy on immunity in preterm infants may derive from the finding that natural killer (NK) cell activity was higher in preterm infants who received massage (11). Studies related to cognitive and neurodevelopment in preterm infants found that preterm infants who received massage had higher cognitive scores at the corrected age of 12 months (12) and higher higher mental development scores at 6 months of age (13). Procianoy compared the neurodevelopment of preterm infants who received massage during hospitalization with that of a control group at 2 years of age and found that preterm infants who received massage therapy had higher Mental Development Index (MDI) scores at 2 years of age (14). Massage therapy for preterm infants can also affect the length of hospitalization of preterm infants, and studies have shown that preterm infants who received massage therapy had a significantly lower length of hospitalization than control preterm infants (15), and infants who received massage were 1.85 times more likely to be discharged from the hospital earlier than control infants (16).

Our search of the relevant literature revealed that previous systematic evaluations and reviews have reported the facilitating effect of massage therapy on the above outcome indicators (weight gain, enhanced immunity, improved cognition, etc.) (17–19); however, there is still controversy as to whether massage therapy can improve mother-infant attachment (20, 21), calorie intake (22), motor function (23, 24), reflexes (23, 25), body temperature (26, 27), and oxygen saturation (24, 28) in preterm infants. Therefore, this study conducted a meta-analysis of the above indicators to assess the efficacy of massage therapy in preterm infants.

## 2. Materials and methods

### 2.1. Operational definitions

1) Preterm infants: Infants born less than 37 weeks or 259 days gestational age (29).
2) Low birth weight: Birth weight less than 2,500 g (30).
3) Massage Therapy: Skin massage is considered a therapeutic touch intervention that involves the planned and purposeful application of tactile stimuli through the person's hands (31). Massage therapy for preterm infants is usually performed using the Field protocol, which lasts for five days, three times a day, one hour after feeding. Massage usually lasts 15 min and is divided into three phases: five minutes of tactile stimulation, five minutes of kinesthetic stimulation, and five minutes of tactile stimulation (26). In addition to this, there are the Vimala protocol (15) and the Vaymal protocol (32). Massage is usually performed by massage therapists, systematically trained researchers, or parents of premature infants (21, 25, 27).
4) Mother-infant attachment: An intimate relational bond formed between mother and infant, specifically, the emotional connection that the mother makes with the infant, and the basis for the infant to build his or her own attachment system (33).
5) Caloric intake: The daily caloric intake of preterm infants per kilogram, usually in kilocalories or joules.

### 2.2. Criteria for inclusion and exclusion

#### 2.2.1. Inclusion criteria

1) Type of participants: Preterm neonates born at a gestation age (GA) under 37 weeks or with low birth weight (<2,500 g); 2) Treatment group: Massage therapy for premature infants; 3) Control group: Preterm infants that are routinely treated; 4) Research type: Randomized controlled trial (RCT); 5) At least one of the following outcome measures was included in the study: mother-infant attachment, oxygen saturation, motor, reflex, temperature, and calorie intake.

#### 2.2.2. Exclusion criteria

1) Retrospective studies, prospective observational studies, narrative review letters, editorials, and commentaries; 2) Literature with identical content; 3) RCTs without a control group; 4) RCTs with a combination of interventions; 5) non-English-language literature; 6) Outcome indicators that could not be extracted or inferred.

### 2.3. Search strategy

We performed a systematic literature search in PubMed, the Cochrane Library, Embase, CINAHL, and Web of Science from their inception until 25 April 2022 for RCTs of massage therapy and routinely treated for preterm infants, using combinations of the following terms: “Massage Therapy,” “Zone Therapy,” “Massage Therapies,” “Zone Therapies,” “Premature Infant,” “Neonatal Prematurity,” “Premature baby,” “Premature births,” and “Randomized Controlled Trial.” In addition, the references in all selected articles were screened for any potential eligible studies. Detailed information on the search strategies are provided in Supplementary Table S1.

### 2.4. Screening and quality assessment of literature

Two reviewers independently evaluated whether each study met the inclusion and exclusion criteria. Disagreements between two reviewers were resolved by discussion with a third reviewer when necessary. Eligibility for inclusion was determined after reading the abstract and consensus was reached through discussion. The extraction of statistics from eligible research was performed by three impartial reviewers who employed standardized tables to ensure consistency. A team of two reviewers was responsible for the data extraction process of the study. To resolve any and all disputes, an additional reviewer was employed as a means of reaching a resolution. The reviewers carefully extracted pertinent information, including interventions and outcome indicators, ensuring a comprehensive analysis of the research. The random effects model was selected for its versatility in accounting for varying degrees of heterogeneity among studies. To combine the results, the effect estimates of the meta-analysis were converted to SMDs and weighted to account for any major study overlaps, ensuring a more accurate and comprehensive summary of the findings. We extracted the baseline data of the articles and the outcome indicators of interest, including the author, title, publication year, general information of the research subjects, and outcome indicators. We created a pre-extraction table taking Valizadeh's article as an example (Table 1).

**Table 1:** Pre-extraction table .

Author	Published year	Design	Country	Study period	Number of participants		Gestational age (weeks)		Sex (males, %)		Birth weight (g)		Intervention methods		Intervention period		Oxygen saturation	
					Intervention group	Control group	Intervention group	Control group	Intervention group	Control group	Intervention group	Control group	Intervention group	Control group	Intervention group	Control group	Intervention group	Control group
Valizadeh (41)	2012	RCT	Iran	2010.1–2011.6	Sunflower oil: 30, Coconut oil: 30	30	Sunflower oil: 30.8 (1.3), Coconut oil: 30.13 (1.41)	30.2 (1.52)	Sunflower oil: 19 (63.3%), Coconut oil: 16 (53.3%)	18 (60%)	Sunflower oil: 1,569.33 (295.24), Coconut oil: 1,519 (333.19)	1,559.33 (360.91)	Sunflower oil: massaged by sunflower oil, Coconut oil: massaged by coconut oil	routine care	15 min once daily for 3 consecutive days	NA	Sunflower oil: 97.54 (2.04), Coconut oil: 96.73 (5.1)	97.09 (2.6)

### 2.5. Assessment of the risk of bias

The methodological quality of RCTs was assessed by the Cochrane “Risk of Bias Assessment Tool” (34). Independent assessments of the risk of bias (ROB) were conducted by two authors (Z.Y and D.C.L). The domains assessed include random number generation, allocation concealment, blinding of intervention and outcome assessors, completeness of follow-up, and other potential sources of bias. Conflicting results were re-evaluated by a third author (C.L.Y). Differences in opinion were resolved by consensus after a team dialogue involving all authors.

### 2.6. Data synthesis

The amalgamation of data was achieved utilizing the complete meta-analysis software program RevMan (version 5.3). We performed statistical analysis to determine the magnitude of change and variability in our outcome metrics of interest. We estimated the SMD and 95% CI for these outcomes. Typically, SMD is used when the studies all assess the same outcome but measure it in a different way or using a different scale. We therefore used the SMD to measure mother-infant attachment and calorie intake. We used the mean difference (MD) to measure motor, body temperature, and reflex. Finally, subgroup analyses of oxygen saturation were performed on different intervention days. The chi-square test and *I^2^* were used to evaluate the statistical heterogeneity between massage therapy effects across the trials. For ease of reporting, we tentatively assigned low *I^2^* values of 25%–50%, moderate *I^2^* values of 51%–75%, and high *I^2^* values of 76%–100% (35).

### 2.7. Design

This meta-analysis adhered to the 2009 Priority Reporting Items for Systematic Review and Meta-analysis Guidelines and was registered on PROSPERO. The registration number was CRD42022337849 (36).

## 3. Results

### 3.1. Characteristics of the included studies

**Figure 1:**
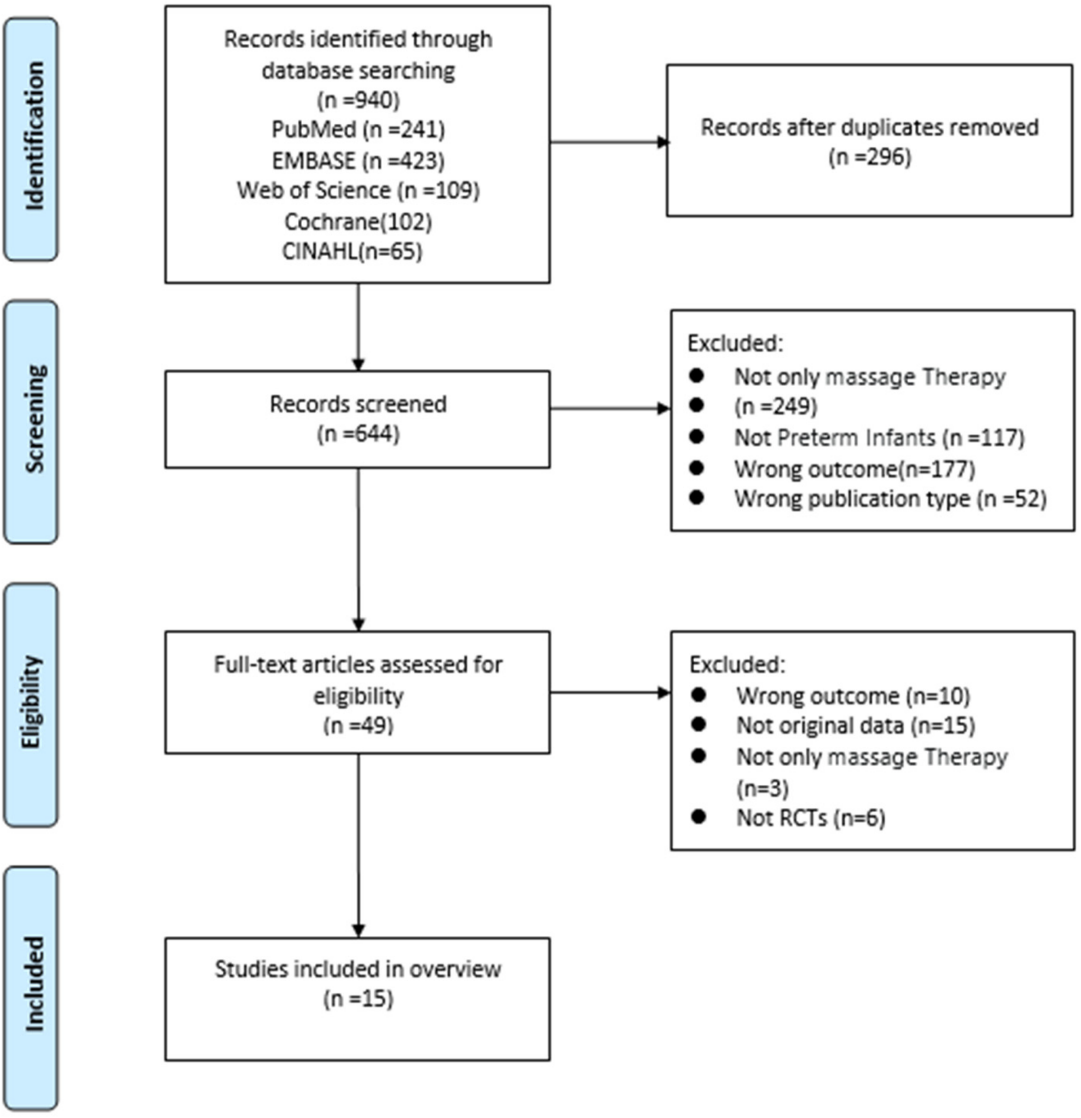
Flowchart of study selection.

By sourcing data from five different databases, a total of 940 studies were identified. After removing 296 duplicates using EndNote software, the remaining 644 articles were reviewed for titles and abstracts, and 595 articles were excluded because the intervention, study personnel, study results, and study type did not meet the inclusion criteria. The full text of the remaining 49 articles was reviewed to assess compliance and inclusion criteria. Finally, 15 studies (15, 21–28, 32, 37–41) were selected as they met all the inclusion criteria and were included in the meta-analyses (Figure 1).

Overall, a total of 15 studies were published between 2001 and 2021, which satisfied the inclusion criteria. In the eligible studies, the sample size ranged from 20 to 90 participants, gestational age from 32 to 37 weeks, and birth weight of participants from 800 to 2000g. Table 2 describe the study and patient baseline characteristics.

**Table 2:** Article baseline data (*n* = 15).

Author	Published year	Design	Country	Study Period	Number of participants		Gestational age (weeks)		Sex (males, %)		Birth weight (g)		Intervention methods		Intervention period	
					Intervention group	Control group	Intervention group	Control group	Intervention group	Control group	Intervention group	Control group	Intervention group	Control group	Intervention group	Control group
Valizadeh (41)	2012	RCT	Iran	2010.1–2011.6	Sunflower oil: 30, Coconut oil: 30	30	Sunflower oil: 30.8 (1.3), Coconut oil: 30.13 (1.41)	30.2 (1.52)	Sunflower oil: 19 (63.3%), Coconut oil: 16 (53.3%)	18 (60%)	Sunflower oil: 1,569.33 (295.24), Coconut oil: 1,519 (333.19)	1,559.33 (360.91)	Sunflower oil:massaged by sunflower oil, Coconut oil: massaged by coconut oil	Routine care	15 min once daily for 3 consecutive days	NA
Elsagh (28)	2019	RCT	Iran	2016.7–2016.9	25	25	34.75 (0.94)		36.40%		2,206.62 (395.71)		Massaged through stroking method	Routine care	15–20 min for 5 straight days	
Seyyedrasooli (32)	2017	RCT	Iran	2,016.5–2,016.6	14	14	30.64 (1.44)	31.14 (1.35)	3 (21.4)		1,346 (290)	1,490 (213)	Massaged by olive oil	Routine care	Two times a day	NA
Diego (27)	2008	RCT	United States	NA	24	24	29.4		54%		1,206		Massage therapy	Routine care	15 min for 5 days	NA
Elmoneim (26)	2020	RCT	Egypt	2014.9–2018.12	30	30	30.7 ± 1.3	31.0 ± 1.3	13 (43.3%)	20 (76.7%)	1,549.8 ± 447.6	1,561.2 ± 347.5	Massage therapy	Routine care	Three consecutive 15-min sessions were performed daily after noon feeding	NA
Hernandez-Reif (39)	2007	RCT	United States	NA	16	16	29.19 (2.04)	29.88 (2.63)	25.00%	31.30%	1,176.56 (244.95)	1,346.25 (360.56)	Massage therapy	Routine care	Received three 15-min massages administered at 9 a.m., 11 a.m., and 1 p.m. each day for 5 consecutive days	NA
Ho (24)	2010	RCT	China	2006.9–2007.6	10	10	30.2 (2.0)	29.6 (2.3)	6 (60%)	7 (70%)	1,104.5 (208.5)	1,093.0 (224.7)	Massage therapy	Routine care	Starting from 34 weeks,recived daily 15 min sessions of massage therapy five times/week for 4 weeks	NA
Arora (23)	2004	RCT	India	NA	Oil massage: 23, Only massage: 23	23	Oil massage: 33.9 (1.7), Only massage: 34.6 (1.1)	34.7 (1.5)	Oil massage: 12 (52.2%), Only massage: 10 (43.5%)	14 (60.9%)	Oil massage: 1,280.2 (170.7), Only massage: 1,298.6 (175.4)	1,327.1 (125.1)	Group 1: oil massage, Group 2: only massage	Routine care	10 min performed four times a day (oil massage group received massage with 10 ml/kg/day of sunflower oil)	NA
Campbell (25)	2021	RCT	United States	NA	8	9	32.4 (1.6)	31.7 (2.1)	5 (62.5%)	4 (44.4%)	2,104 (134.2)		Massage therapy	Routine care	10–15 min of massage three times per day for 5 days	NA
Diego (38)	2005	RCT	United States	NA	16	16	29.8 (3.4)	29.6 (2.7)	25.00%	62.50%	1,091 (193)	1,265 (333)	Massage therapy	Routine care	Three 15-min periods per day for 5 days, 1 h after feeding	NA
Diego (38)	2014	RCT	United States	NA	15	15	29.07 (1.83)	29.40 (1.59)	60%	40%	1,232.67 (186.57)	1,156.53 (180.55)	Massage therapy	Routine care	Three times per day for 5 days	NA
Ferber (22)	2001	RCT	Israel	1996.4–1998.5	21	19	30.90 (1.94)	31.5 (2.22)	NA		1,318 (333.81)	1,527 (34.63)	Massage therapy	Routine care	15 min, 3 times daily at the beginning of three successive hours for 10 days	NA
Gonzalez (15)	2009	RCT	Mexico	NA	30	30	31.4 (2.0)	31.7 (1.8)	26 (86.7%)	21 (70%)	1,235 (243)	1,220 (192)	Vimala massage by their mother or father	Routine care	15 to 20 min twice a day for 10 days	NA
Shoghi (21)	2018	RCT	Iran	NA	20	20	35		15 (75%)	17 (85%)	NA		Massage therapy	Routine care	15 min of massage three times per day for 5 days.	A daily checkup by the doctor, daily weighing, pharmacotherapy, fluid therapy, instruction on proper breastfeeding techniques
Mokaberian (40)	2021	RCT	Iran	NA	20	20	34.40 (1.66)	35.05 (1.39)	NA		1,879.75 (378.41)	1,953.5 (234.39)	Massage therapy	Routine care	Perform the massage in three stages, three times a day, each time for 20 min during a 10-day period	NA

**Table 2:** Continued (outcomes of articles).

Author	Oxygen saturation		Temperature (°C)		Motor		Calorie intake (enteral nutrition)		Reflex		Mother-infant attachment	
	Intervention group	Control group	Intervention group	Control group	Intervention group	Control group	Intervention group	Control group	Intervention group	Control group	Intervention group	Control group
Valizadeh (41)	Sunflower oil: 97.54 (2.04), Coconut oil: 96.73 (5.1)	97.09 (2.6)	NA	NA	NA	NA	NA	NA	NA	NA	NA	NA
Elsagh (28)	First day: 93.26 (1.46), second day: 93.73 (1.66), third day: 93.57 (1.66), fourth day: 93.88 (2.01), fifth day: 94.09 (1.94)	First day: 90.27 (1.12), second day: 90.67 (1.19), third day: 90.21 (0.91), fourth day: 90.70 (1.41), fifth day: 90.47 (0.94)	NA	NA	NA	NA	NA	NA	NA	NA	NA	NA
Seyyedrasooli (32)	First day: 92.07 (0.61), second day: 92.21 (1.05), third day: 93.29 (1.13), fourth day: 95.07 (2.30), fifth day: 93.57 (2.17)	First day: 91.57 (1.08), second day: 92 (1.30), third day: 92.36 (1.64), fourth day: 93 (1.71), fifth day: 93.5 (1.6)	NA	NA	NA	NA	NA	NA	NA	NA	NA	NA
Diego (27)	NA	NA	Pre-during increase: 0.76 (0.41), Pre-post increase: 0.62 (0.43)	Pre-during increase: 0.30 (0.12), Pre-post increase: 0.42 (0.13)	NA	NA	NA	NA	NA	NA	NA	NA
Elmoneim (26)	NA	NA	After massage: 36.8 ± 0.5	NA	NA	NA	NA	NA	NA	NA	NA	NA
Hernandez-Reif (39)	NA	NA	NA	NA	% movement: 1st day = 33.8, last day = 21.7	% movement: 1st day = 24.3, last day = 24.3	NA	NA	NA	NA	NA	NA
Ho (24)	NA	NA	NA	NA	Test of Infant Motor Performance (TIMP) score gain: 34.3 (5.5)	TIMP score gain: 27.2 (5.2)	(J/kg/day) 36 week post-conceptional age (PCA): 570.2 (65.2), 38 week PCA: 534.6 (145.9)	(J/kg/day) 36 week PCA: 559.3 (50.2), 38 week PCA: 605.7 (92.8)	NA	NA	NA	NA
Arora (23)	NA	NA	NA	NA	Oil massage Neonatal Behavior Assessment Score (NBAS): 0 days: 4.1 (0.9);10 days: 4.7 (0.7); Only massage NBAS: 0 days: 3.9 (0.8);10 days: 4.8 (0.4)	NBAS: 0 days: 4.2 (0.7); 10 days: 4.7 (0.5)	NA	NA	Oil massage NBAS: 0 days: 1.0 (1.1); 10 days: 0.6 (0.9); Only massage NBAS: 0 days: 1.5 (2.1); 10 days: 0.6 (0.8)	NBAS:0 days: 1.7 (1.4); 10 days: 0.6 (1.0)	NA	NA
Campbell (25)	NA	NA	NA	NA	Bayley Scale of Infant and Toddler Development-Third Edition (BSID-III): 20.38 (3.84)	18.78 (3.94)	NA	NA	Peabody Developmental Motor Scale-Second Edition (PDMS-2): 2 (0.76)	1.22 (1.3)	NA	NA
Diego (38)	NA	NA	NA	NA	NA	NA	(kcal/kg/d) 111 (11.4)	(kcal/kg/d) 111 (12.4)	NA	NA	NA	NA
Diego (38)	NA	NA	NA	NA	NA	NA	(kcal/kg/d) 114.71 (6.47)	(kcal/kg/d) 118.12 (14.13)	NA	NA	NA	NA
Ferber (22)	NA	NA	NA	NA	NA	NA	(J/kg/day) 707.41 (191.04)	(J/kg/day) 715.76 (183.34)	NA	NA	NA	NA
Gonzalez (15)	NA	NA	NA	NA	NA	NA	(g/d) 1,456 (179)	(g/d) 1,395 (214)	NA	NA	NA	NA
Shoghi (21)	NA	NA	NA	NA	NA	NA	NA	NA	NA	NA	Maternal Attachment Behaviors Scale (MABS): 57.76 (4.20)	MABS: 46.23 (4.35)
Mokaberian (40)	NA	NA	NA	NA	NA	NA	NA	NA	NA	NA	Maternal Postnatal Attachment Scale (MPAS): 72.26(2.96)	MPAS: 64.10(1.87)

### 3.2. Risk of bias

The results of the ROB assessment are reported in Table 3. Six trials used some form of a random sequence generation method. Allocation concealment was not clear in five trials; the risk of selective reporting of results and other biases was low in all trials.

**Table 3:** Overview of risk of bias (*n* = 15).

Studies	Random sequence generation	Allocation concealment	Blinding of participants and personnel	Bliding of outcome assessment	Incomplete outcome data	Selective reporting	Other bias
Valizadeh 2012 (41)	Random blocks method	Low risk	Low risk	Low risk	Low risk	Low risk	Low risk
Elsagh 2019 (28)	Simple sampling	Low risk	Low risk	Low risk	Low risk	Low risk	Low risk
Seyyedrasooli 2017 (32)	Random number table	Low risk	Low risk	Low risk	Low risk	Low risk	Low risk
Diego 2008 (27)	Random number tables	Low risk	Low risk	Low risk	Low risk	Low risk	Low risk
Elmoneim 2020 (26)	Unclear risk	Low risk	Low risk	Low risk	Low risk	Low risk	Low risk
Hernandez-Reif 2007 (28)	Unclear risk	Unclear risk	Low risk	Low risk	Low risk	Low risk	Low risk
Ho 2010 (24)	Unclear risk	Low risk	Low risk	Low risk	Low risk	Low risk	Low risk
Arora 2004 (23)	Random number sequences	Low risk	Low risk	Low risk	Low risk	Low risk	Low risk
Campbell 2021 (25)	Unclear risk	Low risk	Low risk	Low risk	Low risk	Low risk	Low risk
Diego 2005 (38)	Computer-generated randomization list	Low risk	Low risk	Low risk	Low risk	Low risk	Low risk
Diego 2014 (38)	Unclear risk	Unclear risk	Low risk	Low risk	Low risk	Low risk	Risk risk
Ferber 2001 (22)	Unclear risk	Unclear risk	Low risk	Low risk	Low risk	Low risk	Low risk
Gonzalez 2009 (15)	Unclear risk	Low risk	Low risk	Low risk	Low risk	Low risk	Low risk
Shoghi 2018 (21)	Unclear risk	Unclear risk	Low risk	Low risk	Low risk	Low risk	Low risk
Mokaberian 2021 (40)	Unclear risk	Unclear risk	Low risk	Low risk	Low risk	Low risk	Low risk

### 3.3. Heterogeneity and assessment of publication bias

**Figure 2:**
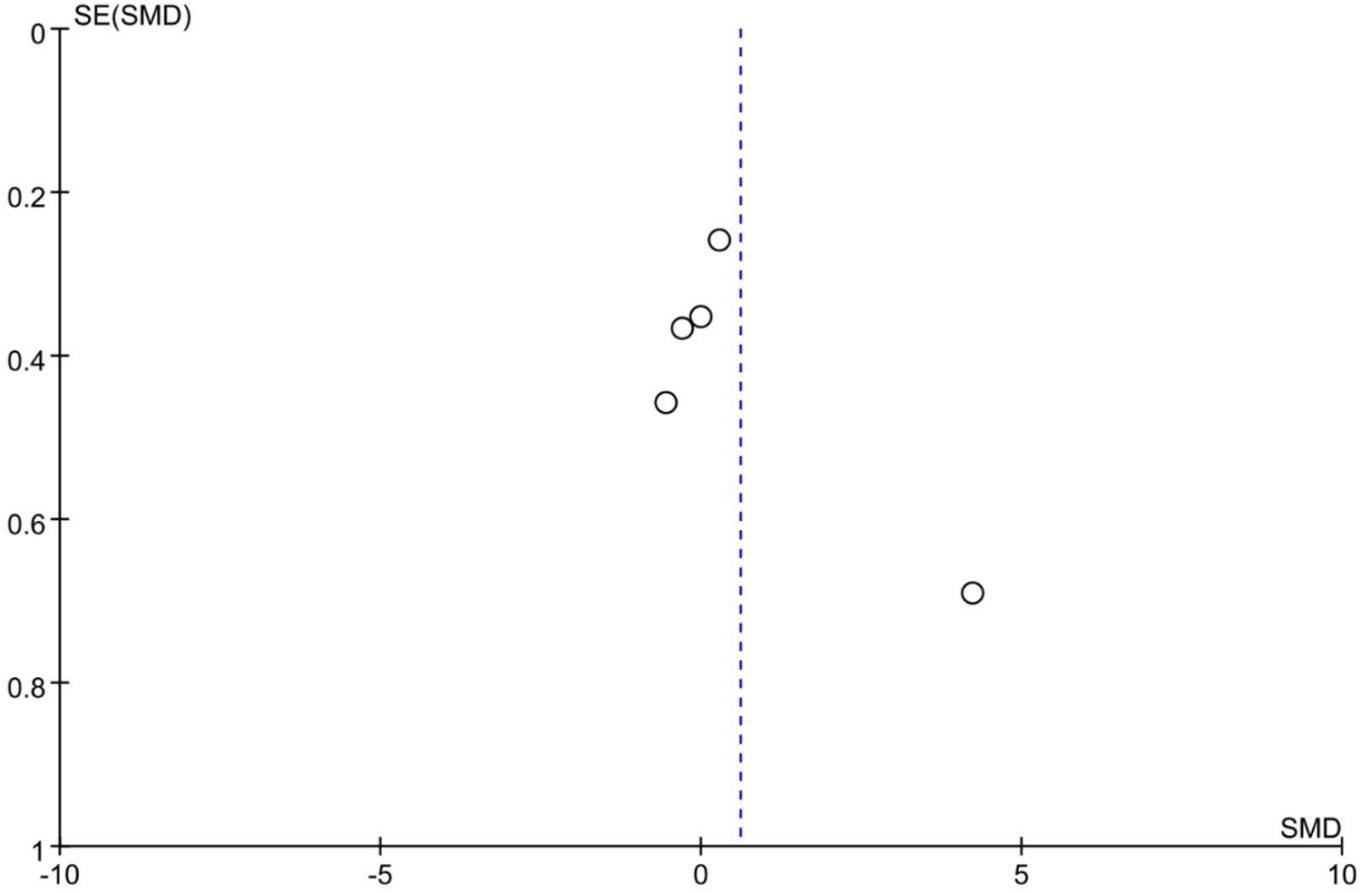
Funnel plot of calorie intake.

**Figure 3:**
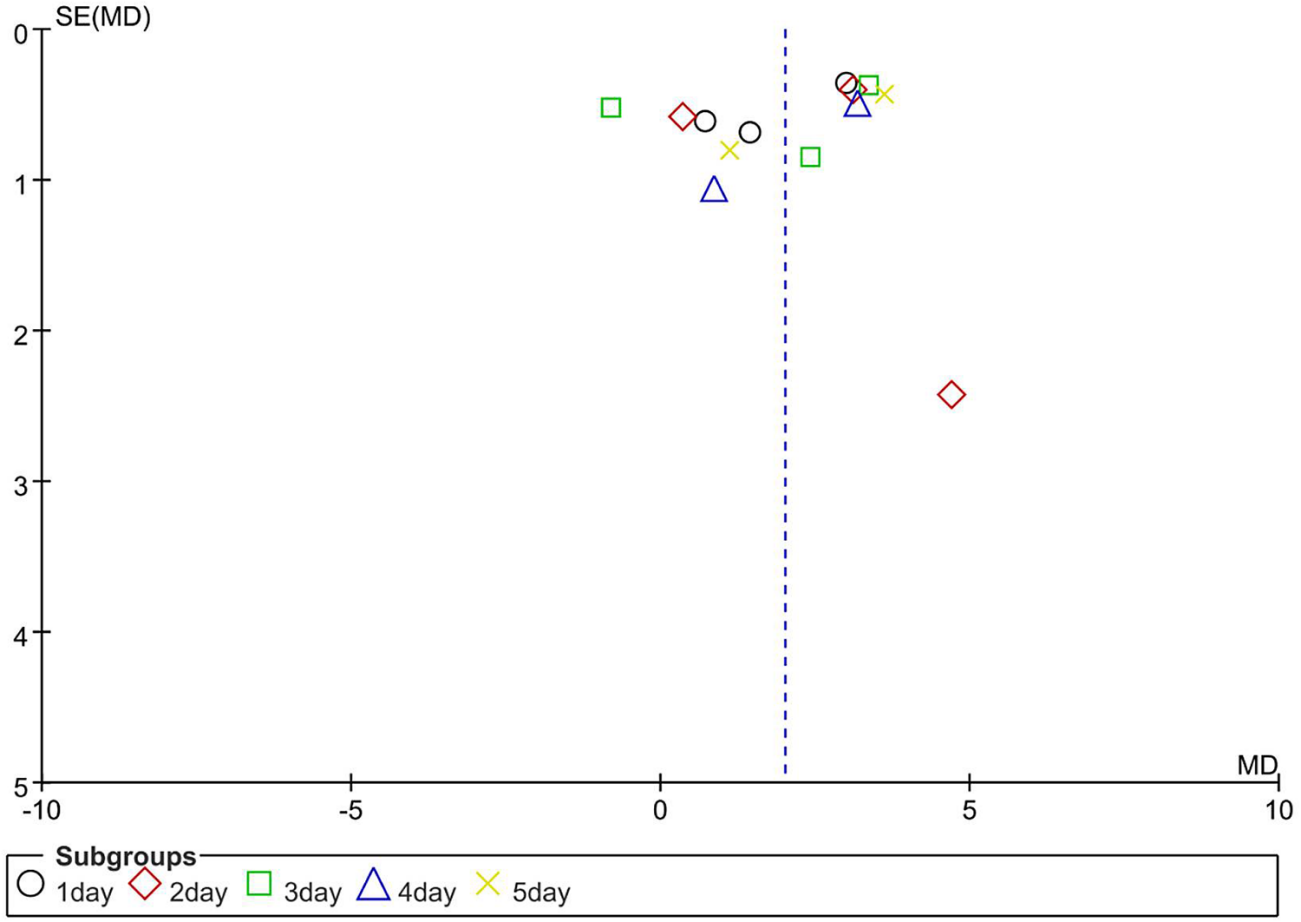
Funnel plot of oxygen saturation.

The table of characteristics of the included studies comprehensively summarizes the study population characteristics, including reflex, motor, maternal-infant attachment, and oxygen saturation in order to assess the inherent heterogeneity. Statistical heterogeneity was estimated using the *I^2^* statistic (42). Assessment of publication bias was conducted utilizing a funnel plot (43). The funnel plot depicted a symmetrical distribution, indicating the absence of publication bias, as exemplified in Figure 2 and Figure 3.

### 3.4. Data synthesis

**Figure 4:**
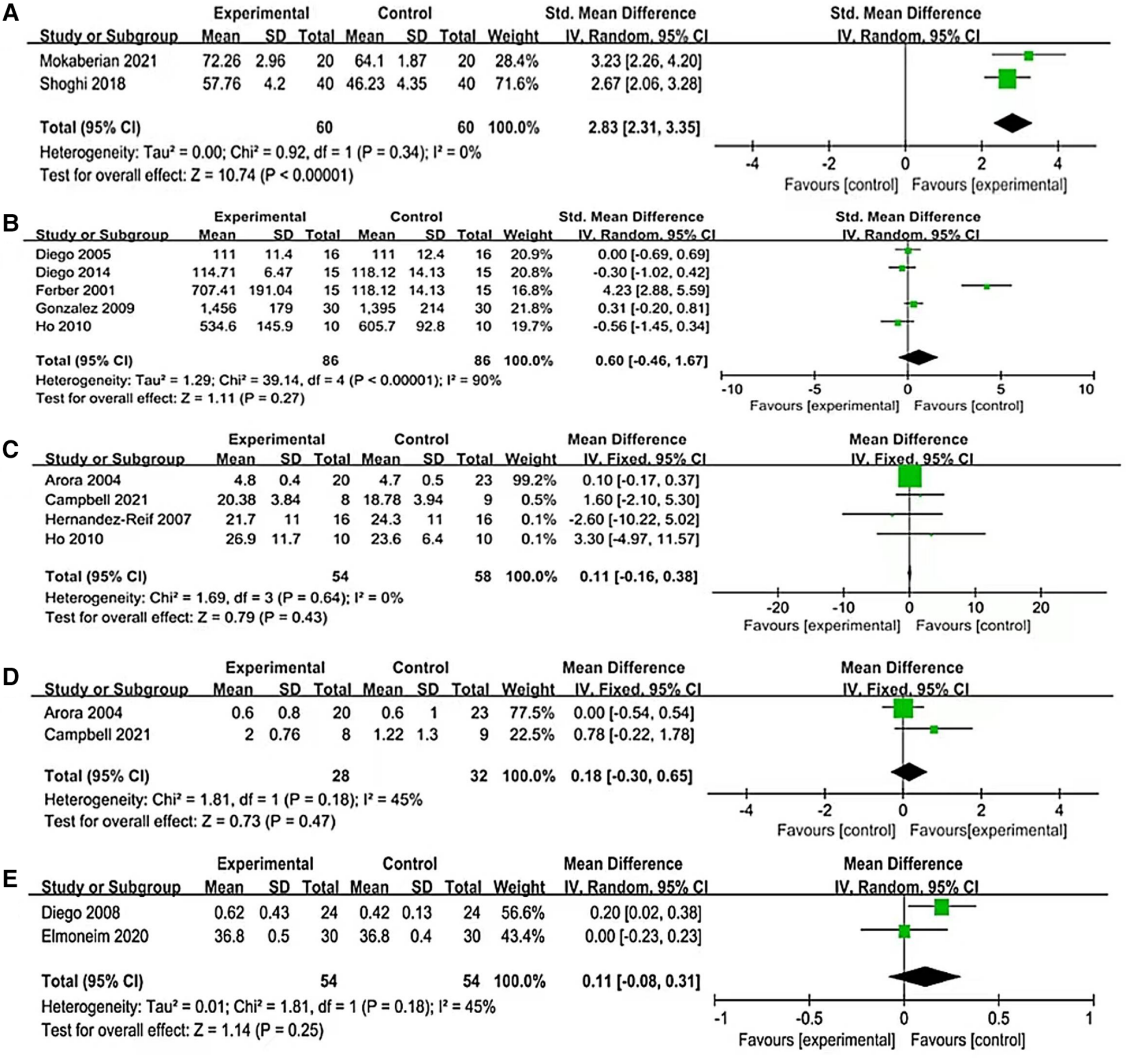
Forest plot of some primary outcomes. (**A**) Mother-child attachment. (**B**) Calorie intake. (**C**) Motor function. (**D**) Reflex. (**E**) Body temperature.

Only two studies have reported the effect of massage therapy on mother-infant attachment. Among them, Mokaberian (40) used the Maternal Postnatal Attachment Scale (MPAS) and Shoghi (21) used the Maternal Attachment Behavior Scale (MABS). The two scholars used different instruments to measure maternal-infant attachment. As shown in Figure 4, there was a significant increase in the level of mother-infant attachment [SMD = 2.83, 95% CI (2.31 to 3.35), *P *< 0.0001]. It was observed that the heterogeneity was relatively minor across these studies. (*I^2 ^*= 0%, *P *= 0.34).

The evaluation of five studies revealed an insignificant impact of massage therapy on calorie intake compared to the control group [MD = 0.60, 95% CI (−0.46 to 1.67), *P *= 0.27], with a high degree of heterogeneity (*I^2 ^*= 90%, *P *< 0.00001). Figure 4 depicts the results of these studies.

Motor function was defined in this study as motor control and organization of posture and movement for functional activities. Four studies reported the effect of massage therapy on motor function in preterm infants. Two of these studies (23, 24) used Neonatal Behavior Assessment Scores (NBAS) and Test of Infant Motor Performance (TIMP) to assess the motor function of preterm infants before and after receiving the intervention. One study used the Bayley Scales of Infant and Toddler Development-Third Edition (BSID-III) (25), while the other study (39) used direct observation of infant limb and trunk movements to assess whether massage therapy improved motor function in preterm infants. Massage therapy had no statistically significant effect on improving the motor function in preterm infants, according to the four studies that evaluated its effectiveness on motor function in preterm infants. [SMD = 0.11, 95% CI (−0.16 to 0.38), *P *= 0.43], with low heterogeneity (*I^2 ^*= 0%, *P *= 0.64), as depicted in Figure 4.

The effect of massage therapy on the reflexes of preterm infants was evaluated in two studies. Of these, Arora's study used the Neonatal Behavior Assessment Scores (NBAS), while Campbell's study used the Peabody Developmental Motor Scales-Second Edition (PDMS-2). The results of the two studies on whether massage therapy affects reflexes in preterm infants were inconsist. As shown in Figure 4, the pooled results indicated that massage therapy had no statistically significant effect on the reflex of preterm infants [MD = 0.18, 95% CI (−0.30 to 0.65), *P *= 0.147], with moderate heterogeneity (*I^2 ^*= 45%, *P *= 0.18).

Two studies have estimated the effect of massage therapy on body temperature. The results indicated that massage therapy had no statistically significant active effect on body temperature, with moderate heterogeneity (*I^2 ^*= 45%, *P *= 0.18), as depicted in Figure 4.

**Figure 5:**
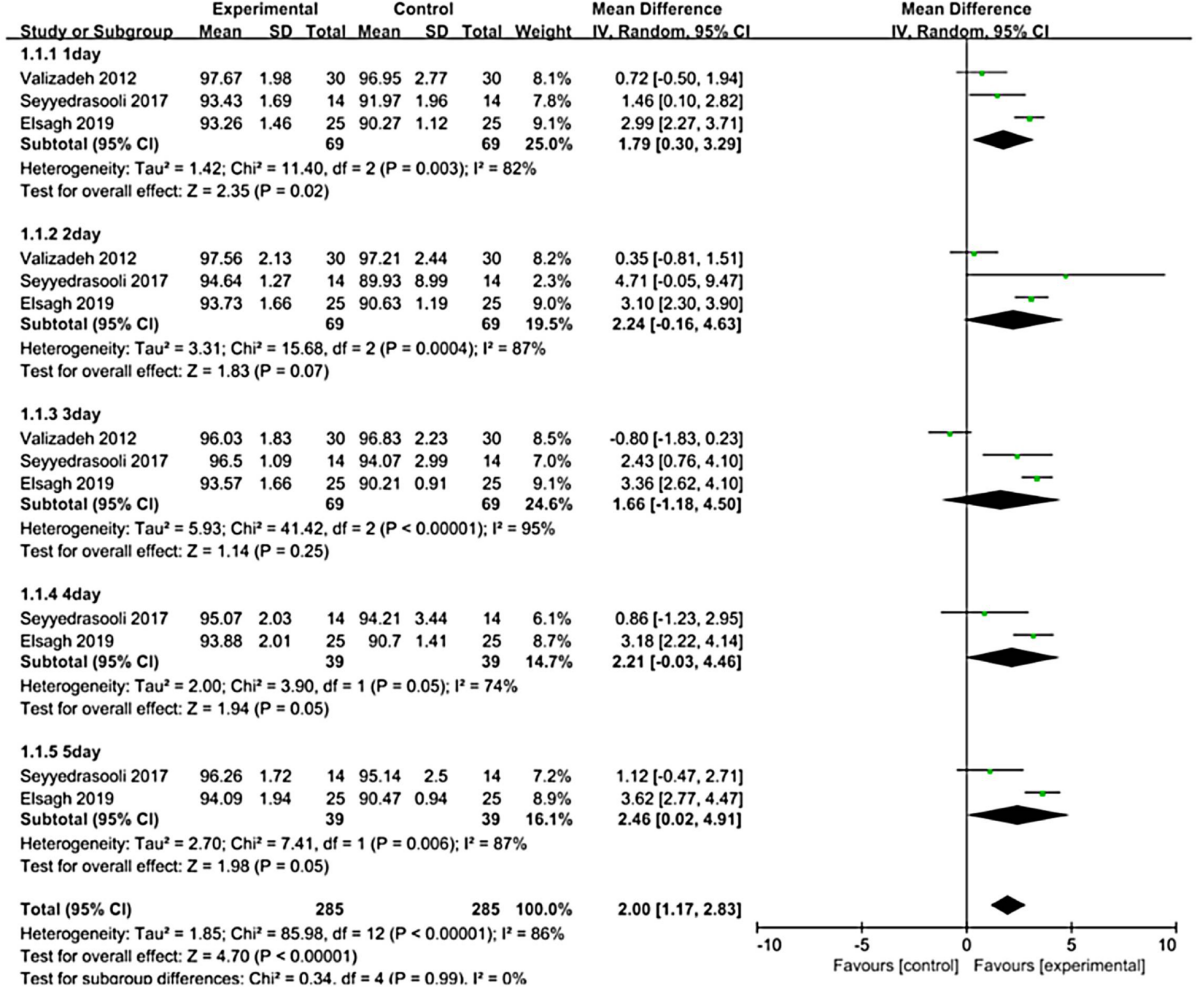
Forest plot of massage therapy vs. standard care on oxygen saturation.

To research the effect of massage therapy on oxygen saturation in preterm neonates, each day of receiving massage therapy was used as the basis for subgroup classification, and the literature results were divided into five subgroups. Subgroup analysis was performed to compare the effect of massage therapy at different time points. Massage therapy showed a significant effect compared to the control [SMD = 2.00, 95% CI (1.17 to 2.83), *P *< 0.00001], and the test for subgroup differences was significant (*I*^2 ^= 86, *P *< 0.00001), as shown in Figure 5.

The findings of the current meta-analysis demonstrated that massage therapy can strengthen the maternal-infant attachment and increase oxygen saturation; however, there was no significant effect of massage therapy on motor, reflex, temperature, or calorie intake.

## 4. Discussion

The effects of massage therapy on preterm infants and their parents have long been of interest, and prior systematic reviews reported the effects of massage therapy on weight gain (44) and maternal and neonatal fatigue (44, 45) in neonatal intensive care units, but did not specifically focus on mother-infant attachment, motor function, reflexes, caloric intake, oxygen saturation levels, or body temperature.

All 15 included studies used massage therapy as an intervention for preterm infants. Twelve of these studies used the Field protocol (21–23, 25–28, 37–41), which consists of massage therapy starting at a corrected gestational age of 35 weeks for five consecutive days, one hour after feeding, three times a day for 15 min. Each treatment was divided into 5 min of tactile stimulation, followed by 5 min of kinesthetic stimulation, and then another 5 min of tactile stimulation (46). One study used a massage protocol developed by the researcher himself (23), the main elements of which were that the massage was first performed on both shoulders, starting at the neck, with the infant in the prone position. The massage was then moved from the upper back to the lumbar region. Subsequently, the two upper and lower limbs were massaged in the supine position. In each area, the massage consisted of 20 gentle strokes. One study used the Vimala protocol (15), which is a massage protocol used primarily in full-term infants, where the infant's torso is divided into six regions: face, upper extremities, chest, abdomen, lower extremities, and back, ending with stretching of the limbs (47). One study used abdominal massage (32), which was performed twice a day for 5 days, at 9 a.m. and 9 p.m., before feeding (48).

This meta-analysis showed a statistically significant effect of massage therapy on maternal-infant attachment; however, due to the small sample size, a positive effect of massage therapy on maternal-infant attachment could not be fully demonstrated. Nevertheless, all of the included studies aimed to demonstrate the effectiveness of massage therapy as an intervention. Effective intervention measures can mitigate maternal anxiety, foster mother-infant attachment, and ameliorate the developmental delay experienced by preterm infants (49, 50). Mokaberian (40) studied the anxiety and association of physical massage with Iranian mothers of preterm infants. The results showed that maternal attachment levels increased significantly in the experimental group. Therefore, massage therapy may be useful as a low-cost, non-pharmacological treatment to improve the psychological state of mothers. Lai's research has also shown that massage has a positive impact on the mother-infant relationship and the neurological development of premature infants (51), consistent with previous research (21, 52).

Developmental care of preterm infants is a current concern. Massage therapy is one of the more effective care strategies (52). This systematic review confirmed that there was a statistically significant difference in oxygen saturation in preterm infants after massage therapy [SMD = 2.00, 95% CI (1.17 to 2.83), *P *< 0.001]. A comparison of the mean daily oxygen saturation of preterm infants who underwent a 5-day massage with control infants showed that there was no statistical difference between the two groups before the start of the massage. On the first day of receiving the massage, the experimental group of infants had an average of 2.99 times higher oxygen saturation than the control infants, and this number increased to 3.62 on the fifth day of receiving the massage (39). Infant massage has been clinically recommended for decades (53). A study by Valizadeh (43) showed that prone position massage in preterm infants was effective in increasing oxygen saturation, which is consistent with the results of the present study.

Some studies have shown that massage therapy can improve reflexes in preterm infants, for example, Solkoff (54) conducted a trial assessing the effect of tactile stimulation using the Brazelton's Neonatal Behavior Assessment Score (NBAS), which demonstrated the beneficial effects of massage in improving neurobehavior in preterm infants. After 10 days of stimulation, the difference in a number of indicators, including habituation and reflexes, was greater than two points, which is inconsistent with the results of the meta-analysis in this article, and there are studies using the same scale to assess whether massage improves neurobehavioral development in preterm infants that have shown no significant improvement in the response to massage therapy in preterm infants (23). Further clinical studies are needed because of the small amount of relevant research.

Caloric intake is usually studied along with weight gain; however, caloric intake does not equate to weight gain, and several randomized controlled trials have shown that massage therapy in preterm infants promotes weight gain but does not significantly affect caloric intake (15, 55). A systematic review also showed that massage in preterm infants did not promote increased caloric intake (56). This is consistent with the results of the present study. However, one study found that preterm infants who received massage had an increase in caloric intake, with no difference in weight gain between the two groups (38). Massage therapy is effective as a low-cost, non-pharmacological intervention to improve physical and motor development in newborns (57), which is consistent with the results of this systematic review. It was concluded that very low birth weight infants who received massage had a higher improvement in motor performance scores (Test of Infant Motor Performance, TIMP) (*P *= 0.043), suggesting that massage may positively affect motor outcomes in a subgroup of very low birth weight infants with lower motor abilities (24). In this study, we found that temperature assessment of preterm neonates randomly assigned to either the control or massage therapy group did not show a statistically significant difference in temperature change between the two groups [MD = 0.11, 95% CI (−0.08 to 0.31), *P *= 0.25], which is in line with the results of a previous study (26). However, a related study concluded that there was no significant difference in temperature between infants who received massage and control infants during the treatment period, but there was a statistically significant difference in temperature changes before and after massage in preterm infants who received massage therapy, with the post-treatment temperature significantly higher than the pre-treatment temperature (*P *< 0.001) (27).

## 5. Limitations

The main limitation of this systematic review is that, in most of the pooled studies, some of the outcomes were not the primary outcomes of interest. Examples include indicators such as weight gain and breastfeeding status. Moreover, in this systematic study, the sample of included studies was small and only two papers reported on maternal-infant attachment among the outcome indicators of interest to us; further studies with expanded sample sizes are needed.

## 6. Conclusion

In conclusion, this systematic review and meta-analysis suggests that preterm infants may benefit from massage therapy because of its potential to improve oxygen saturation, maternal-child attachment, and quality of life in preterm infants. However, due to the limited sample size, further research is needed on the use of massage therapy in the preterm infant population.
