# Supplementary material for: Effects of massage therapy on preterm infants and their mothers: a systematic review and meta-analysis of randomized controlled trials
Source: Front Pediatr. 2023 Aug 31;11:1198730. doi: 10.3389/fped.2023.1198730 (PMC10500070; doi:10.3389/fped.2023.1198730)
Supplement: Supplementary file 1 [file Table1.docx]

Supplementary Table 1. The search strategy, with PubMed as an example

| Database | Search strategy |
| --- | --- |
| PubMed | ((("Massage"[Mesh])OR(Massage[Title/Abstract]ORZoneTherapy[Title/Abstract]ORTherapies,Zone[Title/Abstract]ORZoneTherapies[Title/Abstract]ORTherapy,Zone[Title/Abstract]ORMassageTherapy[Title/Abstract]ORMassageTherapies[Title/Abstract]ORTherapies,Massage[Title/Abstract]ORTherapy,Massage[Title/Abstract]))AND((("Infant,Premature"[Mesh])OR(Infants,Premature[Title/Abstract]ORPrematureInfant[Title/Abstract]ORPretermInfants[Title/Abstract]ORInfant,Preterm[Title/Abstract]ORInfants,Preterm[Title/Abstract]ORPretermInfant[Title/Abstract]ORPrematureInfants[Title/Abstract]ORNeonatalPrematurity[Title/Abstract]ORPrematurity,Neonatal[Title/Abstract]))OR(("Mothers"[Mesh])OR((mothers[Title/Abstract])OR(mother[Title/Abstract])))))AND((random*[Title/Abstract])OR(RandomizedControlledTrial[PublicationType])) |
